# Supplementary material for: Form vision from melanopsin in humans
Source: Nat Commun. 2019 May 22;10:2274. doi: 10.1038/s41467-019-10113-3 (PMC6531428; doi:10.1038/s41467-019-10113-3)
Supplement: Supplementary file 1 — Supplementary Information [file 41467_2019_10113_MOESM1_ESM.pdf]

Form vision from melanopsin in humans

Allen et al.

Supplementary Information

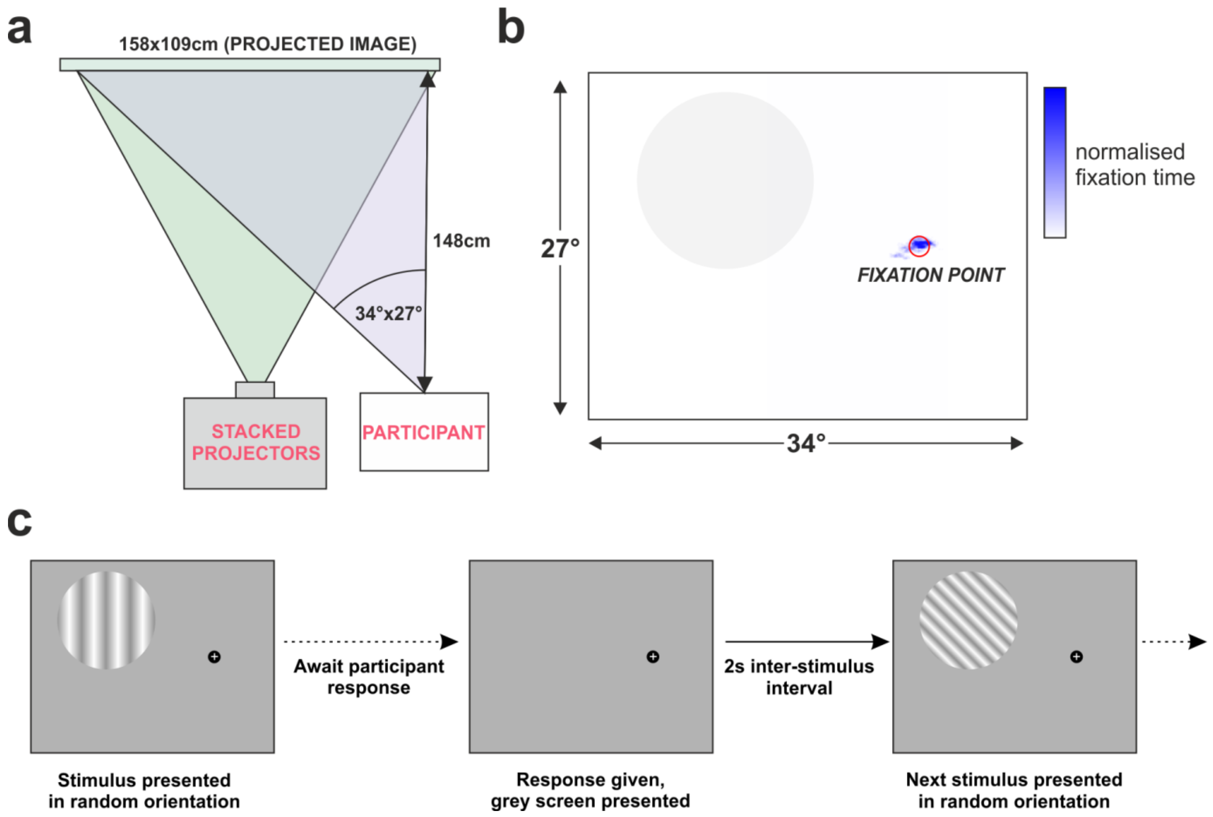

Supplementary figure 1: Procedure overview and presentation details

a) Participants were seated 180cm from the superimposed output of two projectors, which occupied 158x109cm. This resulted in an image occupying 34x27° of visual space. **b)** Participants showed good accuracy in fixation as demonstrated in this representative example, which shows the output of eye-tracking goggles throughout a 30 minute recording (heat map shows normalised fixation time superimposed upon the projector output). **c)** Standard paradigm for assessing detection of static grating stimuli.

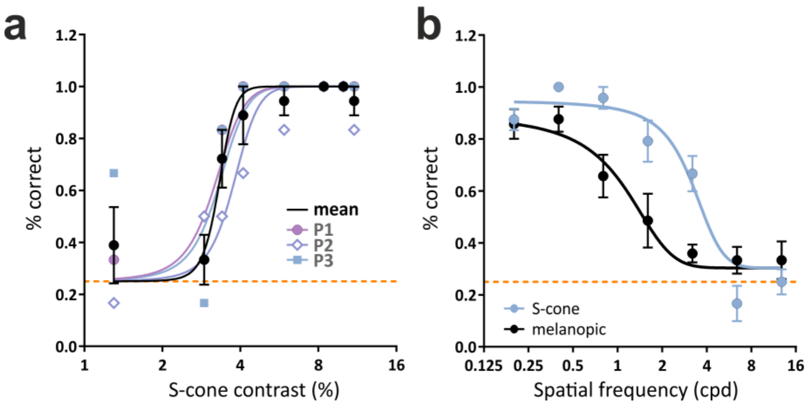

Supplementary figure 2: Spatial frequency tuning of low S-cone contrast stimuli

Of the established visual channels, blue:yellow colour discrimination is most tuned to low spatial frequencies. As an additional confirmation that detection of the ‘melanopic’ gratings did not arise from inadvertent cone contrast we set out to confirm that very low contrast S-cone directed stimuli did not share the spatial frequency preference profile of melanopsin directed gratings. **a)** We described the relationship between proportion correctly identified and contrast for S-cone directed gratings at 0.4 cpd (near peak performance for melanopsin gratings) for 3 individuals (purple, lilac and blue; mean ± SEM response in black). Data are fitted with sigmoidal dose response curves. **b)** Threshold S-cone contrasts for each individual (minimum contrast allowing >80% correct responses at 0.4cpd) were extracted from the data in supplementary figure 2a and used to generate gratings at the full range of spatial frequencies. Graph shows the proportion of correctly identified stimuli for these S-cone stimuli in blue (mean ± SEM) as a function of spatial frequency. Data for individually calibrated ‘melanopic stimuli’ (black; replotted from figure 2) are shown for comparison. Note the rightward shift of the S-cone directed stimulus, indicating that gratings were detectable at higher spatial frequencies. Data are fitted with sigmoidal dose response curves. An F-test comparing these curves finds that they are significantly different ($p<0.05$).

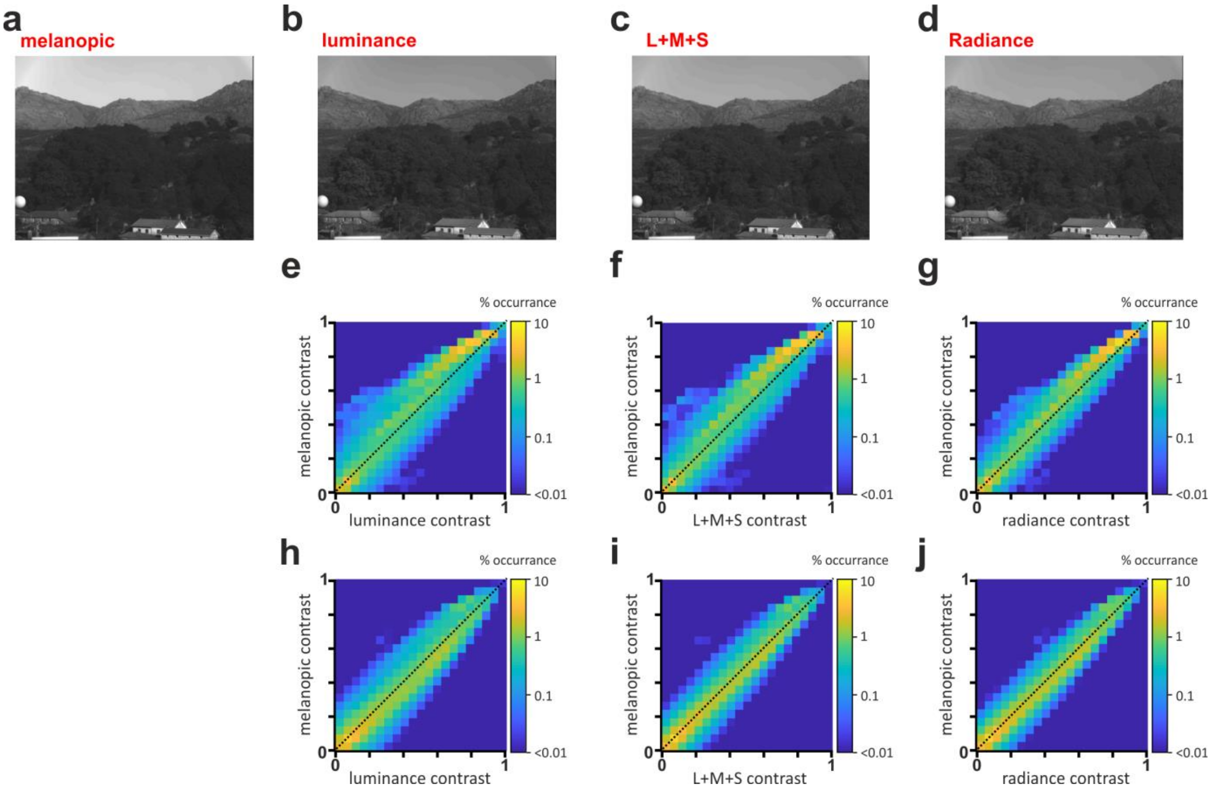

Supplementary figure 3: Correlations between patterns in melanopic contrast and patterns in other measures of light intensity

a-d) Greyscale representations of spatial pattern in melanopic radiance (**a**), luminance (**b**), L+M+S photons (**c**) and total radiance (**d**) for a representative hyperspectral image (HSI)[1]. Scale bars to right: 0 and 1 = lowest and highest melanopic/luminance/L+M+S/radiance pixel, respectively. **e-g)** 2D histogram showing distribution of melanopic radiance contrast vs. luminance contrast (**e**), L+M+S contrast (**f**) and total radiance contrast (**g**) for all pixels pairs in images shown in a-d. Heat maps are scaled to show distributions in contrast as a % of total pixel pairs. **h-j)** As in e-g, but histograms now show distributions in melanopic contrast vs. luminance contrast (**h**), L+M+S contrast (**i**) or total radiance contrast (**j**) for a combination of 13 hyper spectral images.

References

1 Foster, D. H., Amano, K. & Nascimento, S. M. Time-lapse ratios of cone excitations in natural scenes. *Vision research* **120**, 45-60, doi:10.1016/j.visres.2015.03.012 (2016).
